# Influence of Type 1 Diabetes Mellitus on the Ocular Biometry of Chinese Children

**DOI:** 10.1155/2019/7216490

**Published:** 2019-02-14

**Authors:** Ying Xiao, Tao Li, Yan Jia, Shanshan Wang, Chenhao Yang, Haidong Zou

**Affiliations:** ^1^Department of Ophthalmology, Children's Hospital of Fudan University, 399 Wanyuan Road, Shanghai 201102, China; ^2^Department of Ophthalmology, Shanghai General Hospital, Shanghai Jiao Tong University, No. 100, Haining Road, Shanghai 200080, China; ^3^Department of Preventative Ophthalmology, Shanghai Eye Diseases Prevention & Treatment Center, Shanghai Eye Hospital, No. 380 Kangding Road, Shanghai 200040, China

## Abstract

**Purpose:**

To compare ocular biometry between children with type 1 diabetes mellitus (T1DM) and healthy children in China and to determine the correlation of ocular biometry with the glycosylated hemoglobin (HbA1c) level and diabetes duration.

**Methods:**

A case-control study was conducted at Children's Hospital of Fudan University between T1DM children and healthy children. The participants were evaluated for central corneal thickness (CCT), anterior chamber depth (ACD), lens thickness (LT), K1 and K2 keratometry, and axial length (AL); also cycloplegic refraction was performed, and spherical equivalent (SE) was acquired. HbA1c levels of the T1DM cases were obtained.

**Results:**

Fifty-four eyes of 54 children with T1DM and 53 eyes of 53 healthy children were included. The mean age of T1DM group and control group was 10.59 ± 3.40 years and 9.55 ± 1.89 years, respectively, and the differences between age and gender were not significant (*p*=0.052, *p*=0.700). The mean LT in T1DM group (3.49 ± 0.18 mm) was thicker than that in the control group (3.40 ± 0.16 mm) (*p*=0.018), the mean ACD in T1DM group (3.52 ± 0.26 mm) was shallower than that in the control group (3.72 ± 0.26 mm) (*p* < 0.001), and there were no significant differences of CCT, K1, K2, AL, and SE (*p*=0.088, *p*=0.672, *p*=0.821, *p*=0.094, and *p*=0.306, respectively). There was no significant correlation between HbA1c or diabetes duration and ocular biometry.

**Conclusions:**

Thicker LT and shallower ACD occurred in T1DM children rather than age-matched and sex-matched healthy children, but the overall refraction was not affected. HbA1c or diabetes duration was not correlated with ocular biometry in T1DM children.

## 1. Introduction

Diabetes mellitus (DM) may lead to multisystem complications, especially the eyes, kidneys, nerves, heart, and blood vessels [1]. Eye diseases such as cataract, glaucoma, keratopathy, refractive changes, oculomotor nerve paralysis, or diabetic retinopathy (DR) are associated with DM. Although DR is the most noteworthy complication as its threatening outcome is premature blindness, it is rare in children regardless of duration and control of DM. In DM patients, the optical quality might be deteriorated as the tear film, cornea, crystalline lens, and the vitreous are susceptible to hyperglycemia [2, 3]. These changes could be asymptomatic for type 1 diabetes mellitus (T1DM) children, especially when children are young. Axial length, corneal radius of curvature, and lens thickness were the most important determinants of refraction [4]. There is evidence that lens is susceptible in T1DM adults [5, 6]. For growing children, would deteriorations for refractive components affect the refractive development? A previous study indicates that the proportion of myopia is higher in T1DM children aged less than 10 years, but not in older age, and poor glycemic control is not related to higher myopia risk [7]. How does myopia be accelerated in T1DM children? Which components are suffering and leading to this risk, the cornea, the lens, or axial length? Would there be a correlation of the DM condition and the refractive components? Our study aimed to reveal how does T1DM affect children's refractive status.

## 2. Methods

This was a hospital-based case-control study approved by the ethics committee of both Children's Hospital of Fudan University in Shanghai (approval number: No. 01 (2018)) and Shanghai General Hospital (approval number: 2016KY005). This study conformed to the guidelines proposed in the Helsinki Convention. It was a part of the Shanghai Children and Adolescent DM Eye study (SCADE).

Fifty-four eyes of 54 patients with T1DM and 53 eyes of 53 healthy subjects were included in this study. Eligible participants with T1DM were screened from the electronic medical record system of Children's Hospital of Fudan University where they had previously been diagnosed by the endocrine department according to the criteria of the American Diabetes Association [1] and were contacted by telephone to encourage participation. Healthy children were chosen from the ones who had presented to our hospital for routine vision examination. Written informed consent was obtained from each participant at the examination site.

The patients in the T1DM group were under 18 years old and were diagnosed with T1DM at least 1 year before examination. We excluded those with other metabolic disorders (i.e., Prader–Willi syndrome) and contact lens wearers (i.e., orthokeratology lens). Eyes with history of ocular trauma and diseases (i.e., corneal pathology, cataract, glaucoma, optic nerve atrophy, retinopathy, and strabismus) were also excluded. Children in the control group were under 18 years old with normal ocular findings and without systemic problems, and contact lens wearers were also excluded.

Before measurements, a questionnaire was conducted in written form. History of general and eye disease, DM type and complications, onset age and duration, height and weight, blood glucose control method, recent blood glucose level, etc., was reflected in the inquiry.

Examinations were performed by 3 experienced ophthalmologists and 2 optometrists. All of the participants underwent a comprehensive eye examination all in the same day. Before cycloplegia, eye movement and eyelid were checked up; anterior segment was examined using slit lamp biomicroscope; visual acuity (VA) was measured in both eyes using a retro-illuminated logarithmic visual acuity chart; refractive error and K1 and K2 keratometry were measured by an autorefractor (ARK-1; Nidek, Tokyo, Japan); intraocular pressure and central corneal thickness (CCT) were measured by a pneumotonometer (NT-530P; Nidek, Tokyo, Japan); anterior chamber depth (ACD), lens thickness (LT), and axial length (AL) were acquired by IOL (intraocular lens) Master (700; Carl Zeiss Meditec, Dublin, CA); and nonmydriatic fundus photograph was taken by a digital camera (AFC-210; Nidek, Tokyo, Japan). Then, pupil was dilated by 1% cyclopentolate. After that, subjective refraction was performed, and the refraction data was converted to spherical equivalent (SE; SE = sphere power + 1/2 cylinder power). Macular scan was taken by optical coherence technology (OCT) (RS-3000; Nidek, Tokyo, Japan), and fundus blood flow was observed by swept-source OCT angiography (Triton; Topcon, Tokyo, Japan). For all the T1DM patients, fasting venous blood was obtained for determination of serum glycosylated hemoglobin (HbA1c) level.

Statistical analysis of the data was performed with SPSS version 21.0. Mean values and standard deviation (SD) were used for descriptive analyses. Because of the significant correlation between the right and left eyes, only the right eyes were used for the statistical analysis. Kolmogorov–Smirnov test revealed no significant deviation from a normal distribution for all of the test parameters. Independent *t*-test was used to compare age and ocular parameters between the study group and control group. Chi-square test to compare gender, Pearson correlation, and multiple linear regression model were used to determine the correlation between anterior ocular segment biometry and HbA1c level or DM duration. Statistical significance was set as *p* < 0.05.

## 3. Results

The mean age of the T1DM group and control group was 10.59 ± 3.40 years (range 5–17 years) and 9.55 ± 1.89 years (range 5–13 years), respectively; there were 25 male and 29 female patients in the T1DM group and 27 male and 26 female healthy children in the control group, the differences between age and gender of the 2 groups were not significant (*p*=0.052, *p*=0.700). The mean duration of DM was 4.19 ± 2.69 years (range 1–12 years), and the mean HbA1c level at the time of the study was 7.71% ± 2.23% (range 4.6%–14.1%) in the T1DM patients. The mean value of LT in the T1DM group and control group was 3.49 ± 0.18 mm and 3.40 ± 0.16 mm, respectively; the LT was significantly thicker in the T1DM group (*p*=0.018, *α* 0.05, power 0.825). There was also significant difference of ACD (*p* < 0.001, *α* 0.05, power 0.977) between 2 groups, the ACD of control group was 3.72 ± 0.26 mm, which was much deeper than 3.52 ± 0.26 mm of the T1DM group. There were no significant differences of CCT, K1, K2, AL, and SE in 2 groups (*p*=0.088, *p*=0.672, *p*=0.821, *p*=0.094, and *p*=0.306, respectively) (Table 1). Diabetic retinopathy was not seen in any of the patients.

Table 2 shows the correlations among each ocular parameter in 2 groups and the correlations between HbA1c or duration of DM and ocular parameters in T1DM group. Since age was correlated with ACD, LT, AL, SE, HbA1c, and DM duration, age was adjusted besides CCT, K1, and K2 with the multiple linear regression model when there were significant correlations with Pearson correlation. It could be seen that neither HbA1c nor DM duration was correlated with ocular biometry in T1DM group, CCT was not correlated with any of the other parameters in both group, ACD had positive effect on AL in both groups, LT had negative effect on ACD in DM group, and AL had negative effect on SE in both groups.

## 4. Discussion

Overt but asymptomatic changes occur in LT and ACD of T1DM children preceding cataract, glaucoma, DR, and even SE change. Although significant myopia difference could not be read from the SE, underlying mechanism of T1DM is occultly ruining refractive components from our study. Whether these are signs of other complications is still unknown. DR screening examinations for T1DM children are suggested to begin at age 15 years or at 5 years after the diagnosis of DM [3]; however, earlier attention should be paid to refraction. SCADE is a study aiming to investigate the ocular disorders of DM children since January 2018; we will follow up the changing trends in ocular biometry, and the present study provided groundwork and also an inspiration to our research in the future.

The present study showed that the LT was significantly larger accompanied by the ACD decrease than that of the healthy children, which agree with the findings of Uzel et al. [8]. A previous study of internal structure of lens performed with corrected Scheimpflug imaging by Wiemer et al. [9] found that the lens was consisted of three cortical zones and the nucleus; in T1DM patients, all four layers rather than one typical layer of the lens were significantly thicker compared with those of the healthy control subjects, which supports the hypothesis that the thickening of the lens is the result of cellular or extracellular overhydration rather than insulin-induced mitogenesis of the epithelial cells. However, in contrast to T1DM, the lens of type 2 diabetes mellitus (T2DM) patients showed no difference compared with control lens for all layers. This suggests that T1DM and T2DM have different underlying pathophysiologic mechanisms. Does the lens grow larger on account of swelling in T1DM patients? A study used MRI scan by Adnan et al. [10] found the differences in lens shapes between the T1DM and control groups, the diabetes had more rounded shapes with smaller equatorial diameters and greater axial thicknesses; meanwhile, the amplitude of accommodation was smaller, which means the zonules are on greater tension and the ciliary muscles are less contractive on the diabetic eyes. Wiemer et al. [6] also found a more convex lens in T1DM patients that the lens were thicker and both the anterior and posterior radii were smaller. So, the lens becomes rounder rather than larger in T1DM patients. The aforementioned authors also reported a ACD decrease accompanied by the LT grew [6, 8, 10].

On one hand, T1DM had a profound effect on lens; however, CCT, K1, and K2 remained unchanged in T1DM children in the present study. With regard to corneal stroma is a highly hydrophilic structure, it is crucial for epithelium and endothelium to play the role in blocking the penetration of polarized substances from getting into cornea, and the endothelial pumping mechanisms is also vital to maintain corneal dehydration. The DM-caused epithelium/endothelium abnormalities include a decrease in the number of cells, polymorphism, polymegathism, and increase in the cellular coefficient of variation, which affect the barrier functions; hyperglycemia is known to inhibit Na/K ATP-ase-dependent transport of the endothelial cells. It is hypothesized that these changes will lead to corneal hydration and swelling [2, 11]. Some previous studies reported an increased CCT in DM patients than non-DM, regardless of retinopathy status. For example, Suraida et al. [12] found that there was significant mean difference of CCT between non-DM and DM with nonproliferative diabetic retinopathy (NPDR) or no DR in T2DM patients; the NPDR group showed the highest CCT of 529.26 *μ*m, then 524.60 *μ*m for the no DR group, and 493.12 *μ*m for the non-DM group. It differed from the study by Uzel et al., who found patients with juvenile DM had similar CCT, K1, and K2 compared to age- and sex-matched healthy children, the mean CCT value was 542.95 *μ*m and 541.38 *μ*m, respectively [8]. To concur with that, Wiemer et al. [13] measured CCT from 102 patients with T1DM, 101 patients with T2DM, and 69 healthy subjects, and the mean CCT was 0.578 mm, 0.586 mm, and 0.578 mm respectively; no statistically significant difference was found between the 3 groups, whilst the anterior radius and overall corneal power did not differ significantly except for the posterior corneal radii between the 3 groups. Unlike the wide agreement in lens changes of DM patients, it is still controversial whether corneal thickness is affected in DM patients. The discrepancies among different reports could be different devices that are used in CCT measurement, and the HbA1c level may determine the agreement of each pachymetry devices according to Altay et al. [14]. The outcomes in our study were similar to Wiemer et al. and Uzel et al., the mean CCT, K1, and K2 showed no significant difference between 2 groups. Unlike the aforementioned studies, our study showed a marginal significant difference of CCT (*p*=0.088) with mean value 571.02 *μ*m of the control group a little higher than 560.29 *μ*m of the T1DM group; the true reason for this is unknown, and further researches with larger sample size would be needed.

In present study, the AL and SE showed no significant difference between the T1DM group and the control group. Among growing Chinese children from 7 to 14 years, usually a 0.19 mm decrease appears in LT, and increased myopia was related to increases in AL and LT and to decreases in corneal radius of curvature [4]. Given comparatively stable corneal power and AL, a greater LT in T1DM children could be a thinkable risk for myopia. It was concluded by Duke-Elder in 1925 [15] that hyperglycaemia led to myopia, while lowering the blood glucose led to hyperopic. This was proved in the latter ex vivo bovine lens research by Mehta et al. that a trend towards myopia was observed with increasing hyperglycaemia and a hyperopic shift was observed as the glucose return to normal [16]. Nevertheless, there was also reported a myopic shift after a relative hypoglycaemia. Yarbağ et al. [17] found in newly diagnosed T2DM, the average refractive value was +2.50 diopters, and after four weeks' treatment, the average refractive value turned out to be +0.75 diopters as the plasma glucose level went down. So, the question arose whether a decrease in equivalent refractive index of the lens compensated for convex lens shape. Wiemer et al. calculated the equivalent refractive index of the lens in T1DM and T2DM and found a significant decrease in the equivalent refractive index of lenses compared with the control group in T1DM but not in T2DM and combined with more convex lens shape in T1DM and no lens shape change in T2DM, resulting in no lens power change in 2 types of DM [6]. In agreement with that, Adnan et al. revealed a significant decrease in the equivalent refractive index of lenses and no significant change in lens equivalent power in T1DM patients compared with non-DM controls [5]. Lens power could not be directly measured but could be calculated from the refractive indices of the aqueous, lens, vitreous, LT, and the radius of anterior and posterior lens surface [5, 6]. Apparently, LT is one of the determinant factors of lens power yet could not represent lens power. As shown in (Table 2), LT alone was not correlated with SE from our study. It could be deduced that for newly diagnosed DM, hyperglycaemia leads to myopia, this is a transient phenomenon caused by initial lens power increases; however, lens power decreases with the plasma glucose return, and there may be lags for lens power return as the myopic shift was observed after a few weeks' treatment by Yarbag. But the lens status differs for long-term DM with relatively stable plasma glucose. For T1DM, lens refractive index goes down whereas convexity increases; for T2DM, lens shape stays as usual with unchanged lens refractive index as the healthy control. To conclude, lens power increases in uncontrolled hyperglycaemia and the compensation theory exists in T1DM patients. The T1DM children in our study were with at least one year of DM duration, LT increased while SE remained the same compared with healthy controls, and these could be the compensation from lens refractive index.

It was noteworthy that despite LT and ACD significantly changed in T1DM children, no relation was found between the blood HbA1c level or DM duration and LT, ACD, and the other unchanged parameters (AL, SE, K1, K2, and CCT). Our study was in agreement with Uzel et al.'s [8], who found no relationship between LT and the HbA1c level in T1DM children. Several other studies have investigated the effect of DM duration or HbA1c level on ocular parameters. Adnan et al. [5] assessed it in multiple regression fits, and the duration of diabetes contributes to ACD, LT, and lens equivalent refractive index but not contributes to SE, CCT, and lens equivalent power in T1DM adults. In line with Adnan, Wiemer et al. revealed that, in T1DM adults, the duration of DM was found to have significant influences on the ACD, LT, lens refractive index, and lens anterior and posterior curvature, while no associations were found between the duration of DM and the ocular refraction, CCT, and corneal radius; HbA1c was explored to have no significant influence on the various lens and corneal parameters [9, 13]. In the study of Chinese T2DM by Song et al. [18], blood levels of HbA1c were not related to AL, ACD, and corneal radius. It could be concluded that, in T1DM patients but not T2DM patients, lens parameters were sensitive and corneal parameters were apparently stable; for T1DM children, DM duration was too short to have profound effect on lens changes compared to adults; however, for adults, besides DM duration, aging could have an inevitable impact on long-term changes too, and HbA1c level had no effect on ocular parameters of DM patients.

Our study has limitations. This is a cross-sectional data analysis; it only represents ocular status at examining time, because of the sensitivity of lens parameters of T1DM children, fluctuation is possible, and serial ocular biometry measurements are useful in further study. For growing children, ocular biometry is changing along with growth; repeated measurements after a period of time could be helpful to reveal the changing trends besides growth. Larger-scale and multicenter study may be needed to better elucidate how these changes affect the refractive development, and consensus should be made that when shall we start to monitor the refractive development of T1DM children if necessary.

## 5. Conclusions

In T1DM children, we found that LT became larger accompanied by ACD decrease, while the other ocular biometry was apparently unaffected; however, the overall refractive error remained unchanged. We deemed this as a compensation from the lens refractive index. DM duration and HbA1c level did not affect ocular biometry.

## Figures and Tables

**Table 1:** Ocular parameters of T1DM patients and healthy controls.

	DM group (mean ± SD)	Control group (mean ± SD)	*t* value	*p* value
Age, years	10.59 ± 3.40	9.55 ± 1.89	1.963	0.052^a^
M/F, n	25/29	27/26	—	0.700^b^
CCT, *μ*m	560.29 ± 29.29	571.02 ± 31.61	−1.722	0.088^a^
ACD, mm	3.52 ± 0.26	3.72 ± 0.26	−4.104	0.000^a^
LT, mm	3.49 ± 0.18	3.40 ± 0.16	2.422	0.018^a^
AL, mm	23.86 ± 1.36	24.28 ± 1.20	−1.691	0.094^a^
K1, D	42.11 ± 1.69	41.97 ± 1.48	0.425	0.672^a^
K2, D	43.14 ± 1.76	43.22 ± 1.63	−0.227	0.821^a^
SE, D	−1.13 ± 2.45	−1.59 ± 1.96	1.030	0.306^a^

CCT: central corneal thickness; ACD: anterior chamber depth; LT: lens thickness; AL: axial length; K1: flat meridian; K2: steep meridian; SE: spherical equivalent; D: diopters; DM: diabetes mellitus; SD: standard deviation. ^a^Independent *t*-test. ^b^Chi-square test. *p* value < 0.05 significant.

**Table 2:** Correlation coefficients among ocular biometry, HbA1c level, and DM duration.

	ACD (mm)	LT (mm)	AL (mm)	K1 (D)	K2 (D)	SE (D)	HbA1c (%)	DM duration (years)
CCT, *µ*m								
DM	−0.073	0.277	−0.084	0.043	0.039	0.080	−0.117	0.154
Control	0.033	−0.261	0.168	−0.089	−0.011	−0.031	—	—
ACD								
DM		−0.339^*∗*^^a^	0.387^*∗∗*^^a^	−0.059	−0.064	−0.206^a^	0.112	0.004
Control		−0.124^a^	0.308^*∗∗*^^a^	0.128	0.091	−0.214^a^	—	—
LT								
DM			0.011^a^	−0.108	−0.115	−0.045^a^	−0.114	−0.097
Control			−0.149^a^	−0.091	−0.099	0.253^a^	—	—
AL								
DM				−0.364^*∗∗*^	−0.263	−0.863^*∗∗*^^a^	0.010	0.237
Control				−0.375^*∗*^	−0.307	−0.661^*∗∗*^^a^	—	—
K1								
DM					0.945^*∗∗*^	0.304^*∗*^	0.223	0.010
Control					0.939^*∗∗*^	−0.192	—	—
K2								
DM						0.104	0.164	0.050
Control						−0.227	—	—
SE							0.089	−0.064^a^
HbA1c							—	0.234^a^

CCT: central corneal thickness; ACD: anterior chamber depth; LT: lens thickness; AL: axial length; K1: flat meridian; K2: steep meridian; SE: spherical equivalent; D: diopters; DM: diabetes mellitus; HbA1c: hemoglobin A1c. ^*∗∗*^Correlation is significant at the 0.01 level (2-tailed). ^*∗*^Correlation is significant at the 0.05 level (2-tailed). ^a^Beta values by multiple linear regression models, other data are *r* values by Pearson correlation.

## Data Availability

The data used to support the findings of this study are available from the corresponding authors upon request.
